# Right ventricular dysfunction in patients with acute respiratory distress syndrome receiving venovenous extracorporeal membrane oxygenation

**DOI:** 10.3389/fcvm.2023.1027300

**Published:** 2023-05-17

**Authors:** Tyler N. Brown, Thomas V. Brogan

**Affiliations:** ^1^ Pediatric Critical Care Medicine, University of Washington School of Medicine, Seattle Children’s Hospital, Seattle, Washington, United States; ^2^ Department of Pediatrics, University of Washington School of Medicine, Seattle Children’s Hospital, Seattle, Washington, United States

**Keywords:** acute respiratory distress syndrome (ARDS), venovenous extracorporeal membrane oxygenation (ECMO), extracorporeal life support (ECLS), right ventricular failure, acute cor pulmonale (ACP), echocardiography, pulmonary hypertension, pulmonary vascular dysfunction

## Abstract

Acute respiratory distress syndrome is characterized by non-cardiogenic pulmonary edema, decreased pulmonary compliance, and abnormalities in gas exchange, especially hypoxemia. Patients with acute respiratory distress syndrome (ARDS) who receive support with venovenous (V-V) extracorporeal membrane oxygenation (ECMO) usually have severe lung disease. Many patients with ARDS have associated pulmonary vascular injury which can result in elevated pulmonary vascular resistance and right heart dysfunction. Since V-V ECMO relies upon preserved cardiac function, right heart failure has important implications for patient evaluation, management, and outcomes. Worsening right heart function complicates ARDS and disease processes. Given the increasing use of ECMO to support patients with ARDS, an understanding of right ventricular-ECMO and cardiopulmonary interactions is essential for the clinician. A narrative review of the manifestations of right heart dysfunction, as well as diagnosis and management strategies for the patient with ARDS on ECMO, is provided.

## Introduction

Acute respiratory distress syndrome (ARDS) is marked by non-cardiogenic alveolar edema, diminished pulmonary compliance, derangements in gas exchange, and abnormalities in the pulmonary vasculature (1). Pulmonary vascular dysfunction and the associated acute increase in pulmonary vascular resistance (PVR) may negatively affect right ventricular (RV) function potentially resulting in acute cor pulmonale (ACP) (2, 3). Clinical management must address the vascular and gas exchange abnormalities when managing patients with ARDS, especially those supported with venovenous (V-V) extracorporeal membrane oxygenation (ECMO) or extracorporeal life support (ECLS).

The normal pulmonary vascular bed is characterized by low resistance which is lowest at functional residual capacity (FRC) and increases as lung volumes deviate from FRC (4). Consequently, with normal levels of PVR, the RV experiences much lower afterload than the left ventricle (LV). Respiratory diseases can affect this relationship by causing vasoconstriction secondary to alveolar hypoxia and hypercarbia, acidemia, release of inflammatory mediators, and decreased lung compliance (1, 5, 6). Furthermore, pulmonary inflammatory endothelial damage, microvascular thrombosis, and vascular remodeling can also affect PVR and RV afterload (7).

The vascular manifestations of lung disease have important effects on cardiac function. The relationship between ARDS and cardiac dysfunction has been well documented (1–11). The RV is subjected to increased afterload during acute lung disease potentiating RV dysfunction. There is a high incidence of RV failure in patients with ARDS, with rates ranging from 10% to 50% (6–11). As the source of LV preload and role of the RV in ventriculoventricular interactions, RV dysfunction can result in decreased cardiac output and vascular congestion which may further compromise organ function.

Given the increasing use of ECMO to support patients with ARDS, an understanding of right ventricular-ECMO and cardiopulmonary interactions is essential for the clinician. This review seeks to provide a broad overview of RV physiology and the role of the right ventricle in V-V ECMO. A narrative review of the manifestations of right heart dysfunction, as well as diagnosis and management strategies for the patient with ARDS on ECMO, is provided.

## Right ventricular physiology, ventriculoventricular interaction, and right ventricle-pulmonary interactions

Historically, the RV has been viewed as the lesser of the two ventricles with the less essential role of sending blood to the lungs while the LV perfuses the entire body (12). This perception has resulted in proportionally greater research into the LV than the RV (13–15). While oversimplified, this understanding of the RV is not without some physiologic veracity. The ability to palliate congenital cardiac dysmorphisms such as hypoplastic left heart syndrome (HLHS) down a pathway with a single systemic ventricle highlights the ability of the cardiovascular system to temporarily function without a pump within the pulmonary circulation (16, 17). Despite the historical designation as the lesser of the ventricles, there is a growing understanding of the essentiality of the RV and its unique physiologic factors that affect the entire cardiac functions.

The RV has a complex structure determined by a unique myofiber arrangement and interactions with the LV (14, 18). The deeper muscle layers of the RV predominate and are arranged longitudinally from the cardiac base to the apex resulting in shortening parallel to the long axis of the RV. The more superficial layers of myofibers are arranged helically along the short axis of the ventricle with contraction resulting in decreased cross-sectional area (torsion) of the ventricle. The combined effect of contraction in these two different myofiber planes results in greater longitudinal than helical shortening. While often considered as separate entities, the right and left ventricles cannot be divorced from one another either anatomically or functionally. Anatomically, this interdependence can be seen in two areas. The RV and LV share a significant portion of myofibers that form circumferential tracts around both ventricles (19). These tracts allow for simultaneous and coordinated biventricular contraction. Furthermore, contraction of the LV produces traction on the RV free wall hinge points (where the RV free wall joins the interventricular septum) that complements RV myofiber contraction (14). Additionally, there is significant ventricular interplay related to the interventricular septum itself. In the healthy state, the pressure difference between the ventricles combined with the less compliant and thicker LV myocardium results in the interventricular septum assuming a convex geometry. With coordinated biventricular contraction, accentuation of interventricular pressure differences and thickening of the septal myocardium results in further compression of the RV cavity, thus supplementing RV contraction (18). The practical pathologic implication of the cross-ventricular myofiber arrangement and effects of the interventricular septum is that dysfunction of one ventricle can induce dysfunction within the other.

The physiology of RV contraction also cannot be isolated from the pulmonary vasculature. In the healthy state, the RV ejects into a low-resistance, highly compliant pulmonary vascular system. Accordingly, when compared to the LV, the RV exhibits a shorter isovolumetric contraction time, earlier ejection, and lower peak systolic ventricular pressure. Additionally, the phenomenon of a hangout interval—continued ventriculoarterial ejection extending beyond cessation of ventricular contraction—occurs exclusively within the right heart (20). This phenomenon is related to the exceedingly low vascular resistance and negative intrathoracic pressure that are characteristics of the RV-pulmonary vascular system. When compared to the LV, the thinner-walled RV is exquisitely sensitive to alterations in afterload (changes in PVR) with increases causing ventricular dilation and precipitous drops in stroke volume (21, 22). Furthermore, as demonstrated in an elegant study by Brookes et al., acute dilation of the RV results in impaired LV contraction likely related to altered geometry and dysfunction of shared myofibers (18, 23). Taken together, the RV should be seen as uniquely suited to the normally low-resistance state of the pulmonary vasculature and that any alteration of this typically low pressure system may result in RV dysfunction with subsequent impairment of normal biventricular function.

## Right ventricular dysfunction in patients receiving ECMO for ARDS

Patients with ARDS present a model for perturbation of typical RV-pulmonary interactions. Studies of the influence of RV failure on mortality in adults with ARDS have demonstrated conflicting results. Some studies show higher mortality (7–9), while others show no difference (10, 11). However, a large meta-analysis of 9 studies including 1,861 patients with ARDS found that RV injury (defined as RV dysfunction, RV dysfunction with hemodynamic compromise, RV failure and ACP) occurred in 21% of ARDS patients (3). In this meta-analysis, the included studies used varied modalities to assess RV injury including pulmonary artery catheter (PAC) (2 studies), trans-thoracic echocardiography (TTE) (4 studies), trans-esophageal echocardiography (TEE) (1 study), or either echocardiographic modality (2 studies). The analyzed studies were generally performed after widespread adoption of lung-protective ventilation which has also been shown to be protective of the RV. In the pooled meta-analysis, RV injury was associated with a significantly higher risk of short-term mortality (OR: 1.48, 95% CI: 1.14–1.93, *p* = 0.003, *I*^2^ = 0%) and overall mortality (OR: 1.45, 95% CI: 1.13–1.86, *p* = 0.003, *I*^2^ = 0%). These data were supported by another study of 752 patients with ARDS in which a PaO_2_/FiO_2_ ratio < 150 mmHg and a PaCO_2_ ≥ 48 were found to be independent factors associated with ACP (6).

While ARDS is associated with RV dysfunction, this relationship is further exacerbated in the patient with ARDS supported on V-V ECMO. Cardiac dysfunction in patients with ARDS receiving ECMO appears to be strongly related to RV dysfunction. One study compared patients who had clinical evidence of cardiac dysfunction at cannulation with those who did not (24). Of the 92 patients on V-V ECMO, those who required vasoactive support had lower pH and PaO_2_ with higher lactate levels. Echocardiography revealed a higher incidence of RV dysfunction (39%) and biventricular failure (13%) in these patients. Interestingly, there was an increase in the proportion of patients with RV dysfunction following cannulation. In addition to BMI and PO_2_, RV dilatation was a significant predictor of mortality. Another study evaluating 121 patients with pre-ECMO hyperlactatemia showed that RV dilation before ECMO support was an independent risk factor for death (OR: 0.239, 95% CI: 0.101–0.561, *p* = 0.001) (25). Highlighting the relationship between RV dysfunction and systemic perfusion abnormalities, in this study lactate levels correlated with RV dysfunction as measured by tricuspid annular plane systolic excursion (TAPSE) (25).

Adult patients with ARDS who receive V-V ECMO support have significant rates of RV dysfunction. In 46 adults supported with V-V ECMO for ARDS, 60% of the patients had RV hypertrophy (RVH) at cannulation (26). Furthermore, all those with initially normal RV thickness developed RVH during their ECMO course. Duration of mechanical ventilation prior to ECMO did not differ between the two groups. A minority (17%) of the patients received prone positioning before cannulation. There was no difference between groups in mortality, which was 30%. Unfortunately, the authors did not report the evolution in the RVH after discharge from the ICU. In a similar study evaluating 130 adults with ARDS, 80% had RVH before cannulation which increased to 90% after cannulation (27). Based on either quantitative or qualitative measurement, RV dysfunction was found in from 1/3 to 2/3 of patients before ECMO and between 1/2 and 2/3 after cannulation. The findings of both studies are limited by their small number and single center, retrospective design.

Early post-cannulation RV dysfunction appears to be an important phenomenon in ARDS patients. A retrospective study of 64 patients evaluated for RV dysfunction on ECMO (defined as RV dilation plus septal wall motion abnormalities on the first post-canulation echocardiogram) (28). In this study, RV dysfunction was associated with decreased survival to ECMO decannulation (45% vs. 83%) and hospital discharge (32% vs. 64%). Regression analysis showed that absence of RV dysfunction and small LV were associated with survival to decannulation (OR: 6.95 95% CI: 1.87–19.28) and hospital discharge (OR: 1.292, 95% CI: 1.015–1.645, *p* = 0.038). Systematic detailed studies of RV dysfunction in children with ARDS supported with ECMO are lacking but case series have reported ACP in pediatric patients with acute respiratory failure on V-V ECMO (29).

In addition to RVH and RV dilation, ARDS patients may demonstrate pulmonary hypertension. In 74 patients with ARDS supported with V-V ECMO, the pre-ECMO echocardiogram was normal in only 34% of patients while 43% had isolated pulmonary hypertension (PH) and 23% had PH with RV dilation (30). Additionally, almost 20% of patients had LV dysfunction as demonstrated by a reduced LV ejection fraction. Total ICU mortality was 41.8%. Regression analysis showed that RV dilation and BMI were associated with mortality.

## The effect of ECMO on RV function

Because gas exchange occurs primarily via the extracorporeal circuit in patients on V-V ECMO, extracorporeal support provides the opportunity to institute protective or ultraprotective lung protective strategies for mechanical ventilation. These ventilatory approaches are characterized by low tidal volumes (*V_t_*) and respiratory rates. When appropriately targeted to maintain the lungs at or near FRC, these strategies can help decrease PVR and promote normal RV function (31). Furthermore, by raising the mixed venous oxygen level (SvO_2_), V-V ECMO may result in a decrease in PVR through reversal of hypoxic vasoconstriction (32). This phenomenon is supported by extrapolation of findings in the management of pulmonary arterial hypertension (33). Given the exquisite sensitivity of the RV to changes in PVR, the practitioner must be vigilant to avoid significant atelectasis or consolidation while implementing lung protective ventilation strategies. In this scenario, pulmonary consolidation results in vascular compression, diminished cumulative pulmonary capillary cross-sectional area, and the associated increase in PVR.

In patients with ARDS, ECMO can correct abnormalities in ventilation and oxygenation with an immediate decline in pulmonary artery pressure and increase in cardiac contractility (34). In one small study, 13 adults with ARDS were cannulated onto V-V ECMO and pulmonary artery pressure and cardiac index (CI; thermodilution method) were monitored via a Vigilance II monitor (Edwards Life Sciences, Irvine CA) utilizing a pulmonary artery catheter (35). Multiple parameters including mean pulmonary artery pressure (mPAP) and CI were monitored prior to and after cannulation at multiple timepoints. There was a decline in mPAP within 30 s of ECMO commencement. The fall in mPAP was associated with the drop in PaCO_2_ and an increase in SvO_2_. The decrease in mPAP was mirrored by an increase in CI and a drop in central venous pressure (CVP).

Noninvasive measurement of cardiac function has similarly demonstrated that cardiac function generally improves following initiation of ECMO. One multicenter study of 675 patients with ARDS related to SARS-CoV-2 infection utilized echocardiography to evaluate function and found that while LV dysfunction was global, RV dysfunction appeared to be related to alterations in RV afterload from mechanical ventilation, hypercapnia and pulmonary emboli (36). Another study utilizing echocardiography examined 7 patients with pre-ECMO ACP and found significant improvement in RV function (as measured by elevated pulmonary artery Doppler, reduced RV fractional area change, and RV free wall longitudinal strain) occurring within 24 h of cannulation (37). This amelioration of RV dysfunction was attributed to correction of hypoxemia and hypercapnia related to decreases in ventilatory support and associated intrathoracic pressure and RV afterload.

In contrast to V-V ECMO, venoarterial (V-A) ECMO and the hybrid cannulation strategy of venovenoarterial (V-VA) ECMO directly decrease preload to the right heart and provide direct cardiac support (38). The V-A strategy is often used in patients with evidence of more severe cardiac dysfunction (38). Distinct from V-A ECMO, the V-VA configuration also acts like V-V ECMO and provides well oxygenated and ventilated blood to the pulmonary circulation. This strategy thereby preserves the pulmonary vasodilatory effects of a higher SvO_2_ while also reducing the volume load of the RV and is often considered when patients appear inadequately supported by V-V ECMO (39). In one small study, 30-day mortality was lowest in ARDS patients receiving V-VA support as compared to V-V and V-A ECMO modalities.

## Diagnosis of right ventricular dysfunction

### Echocardiography

Echocardiography is the most widely employed modality for monitoring cardiac function (5, 40). In patients supported on ECMO, echocardiography typically represents the only feasible non-invasive modality for assessing cardiac function. Echocardiography allows for bedside assessment with a reasonable degree of reproducibility and interobserver reliability (41–43). While ubiquitous, the use of echocardiography can be limited by numerous factors. These include patient factors such as cardiac position, patient position, and impaired sonographic windows secondary to body habitus or lung artifact (34). Additionally, echocardiography remains a highly user-dependent modality in which sonographer training and experience can affect image acquisition (44). Beyond these technical challenges, this modality is also susceptible to the intrinsic limitations of RV anatomy. The LV, which has a relatively uniform bullet-shape, can be geometrically modeled with relative ease allowing for accurate estimations of ventricular volume to be made using only a few echocardiographic data points. The presence of coarse intraventricular trabeculations, variable pyramidal shape, and significant change in conformation throughout the cardiac cycle make estimating RV volumes more challenging. By comparison to magnetic resonance imaging (MRI), 2-dimensional (2D) echocardiographic estimations of RV volume are very poor (45). Consequently, determination of RV size by 2D echocardiography has largely been dependent on the skill and experience of the interpreting echocardiographer. The advent of 3-dimensional (3D) echocardiography has provided improved capacity for objective bedside estimation of RV volumes, although significant intermodal variability persists (46, 47). One non-volumetric evaluation of RV dilation of clinical significance is the ratio between RV end-diastolic area and LV end-diastolic area (RVEDA/LVEDA). This ratio can be obtained by measuring the area of the RV and LV from an appropriate apical four-chamber view. Acute cor pulmonale has been associated with several echocardiographic findings including the presence of paradoxical septal wall movement with RV dilatation (RVEDA/LVEDA >0.6) (48).

Similar to the challenges affecting assessment of RV volume, deriving objective measures of RV function has been difficult. Measures such as ejection fraction (EF), shortening fraction (SF), and fractional area change (FAC) rely upon assumptions of uniform contraction and optimal imaging planes, these metrics are susceptible to the same limitations as 2D volumetric analysis (18). Interestingly, when compared to the gold standard of MRI-derived measurements of RVEF, subjective evaluation (“eyeballing”) of RV function was >95% sensitive for detecting reduced function (RVEF <50%) but <56% specific with sensitivity and specificity improving with the degree of evaluator experience (49). The difficulties in deriving direct measures of RV function have led to the development of surrogate functional indices.

The most common echocardiographic estimates of RV function include TAPSE, RV-S', RV myocardial performance index (MPI, Tei Index), and *dP*/*dT*. Both TAPSE and RV-S' are measures of longitudinal RV contraction that can be obtained with relative ease. Both only measure longitudinal contractility and are limited by their high dependence on obtaining an optimal and appropriate image (49). MPI is derived from the sum of isovolumetric contraction time and isovolumetric relaxation time divided by ejection time (50), while *dP*/*dT* uses the tricuspid regurgitant jet velocity to assess the change in RV pressure (*dP*) over time (*dT*) (51). Both MPI and *dP*/*dT* are calculated measures of function that require analysis of a specific Doppler patterns that can be technically difficult to obtain. While each measure provides an incomplete assessment of function, growing evidence suggests that the combination of subjective and objective measures of function improves diagnostic accuracy (49).

Broader implementation of 3D echocardiography may improve the accuracy of quantitative measures of RV function. While 3D echocardiography technology has been widely adopted in many technologically advanced nations, implementation of 3D echocardiography protocols for RV function has been limited by the time-consuming and user-dependent nature of most software programs. The development of new machine learning-based programs may allow for broader clinical adoption (47).

The concept of right ventricular-pulmonary artery (RV-PA) uncoupling deserves special attention. Derived from research in the pulmonary hypertension population, RV-PA coupling refers to the dynamic relationship between RV contractility and the afterload against which the RV works (52). In its truest definition, RV-PA coupling is the ratio between RV end-systolic elastance (*E*_es_) and pulmonary arterial elastance (*E*_a_) with a *E*_es_/*E*_a_ ratio of 1.5–2 (53). In the normal physiologic state, RV contractility should match RV afterload (RV-PA coupling) but in the diseased state, RV afterload can increase disproportionately to RV contractility (RV-PA uncoupling). While RV-PA coupling is most accurately assessed through pressure-volume loops created using invasively derived measures, non-invasive echocardiographic surrogates have been developed (54). With this approach, a ratio of TAPSE to pulmonary artery systolic pressure (PASP) can be used with values less than 0.36 mm/mmHg representing significant RV-PA uncoupling and a threshold value of 0.31 mm/mmHg being associated with a nearly 90% sensitivity for detecting RV-PA uncoupling when compared to the invasively-derived gold standard (55).

Several studies have applied the concept of RV-PA uncoupling to the ARDS and ECMO populations. In one study of 94 patients with ARDS secondary to SARS-CoV-2 infection, early and substantial RV-PA uncoupling was described (56). In this study, the TAPSE/PASP ratio—like the PaO_2_/FiO_2_ ratio—was associated with increased mortality risk. In another study of 79 adults supported with V-A ECMO for cardiogenic shock, evaluation of RV-PA uncoupling was superior to other echocardiographic parameters for predicting successful weaned from ECMO (57). Further specific studies evaluating RV-PA uncoupling in patients with RV dysfunction supported with V-V ECMO secondary to ARDS are needed.

### Pulmonary artery catheter

Pulmonary artery catheters (PAC) provide direct measurement of pressures relevant to RV function including CVP or RV end-diastolic pressure (RVEDP), RV systolic pressure (RVSP), and pulmonary artery pressure. Furthermore, cardiac output can be derived by thermodilution (assuming a lack of intracardiac shunting). Additionally, a PAC allows for repeated measures and real-time feedback on the effects of various interventions. However, the invasive nature of PACs and the attendant risks have resulted in declining use (40).

## Care of the patient with ARDS on V-V ECMO

### General principles

Since it does not provide direct cardiac support, the goals of caring for a patient on V-V ECMO should include providing adequate gas exchange and optimizing RV function while limiting factors that will increase RV stress and strain (Table 1) (34, 40, 58). Assiduous attention to the basics of patient care undergirds all ECMO care but detailed discussion on basic care is beyond the scope of this review. Optimizing oxygenation, ventilation and serum pH are of primary importance. Fluid overload must be avoided if possible and addressed if already present at the time of ECMO cannulation. Of note, longer duration of mechanical ventilation prior to ECMO may contribute to greater mortality (59).

**Table 1:** Approaches to the management of right ventricular dysfunction in patients with ARDS.

General therapy	Problem	Approach	Goal
	Hypotension	Vasopressor therapy: – Norepinephrine – vasopressin	Coronary perfusion pressure – high dose NE (>0.5 µg/kg/h) can increase PVR and cause tachycardia
	Fall in cardiac output with RV/PA uncoupling	Inotropic therapy – Dobutamine (potent chronotrope) – Milrinone (vasodilator, contraindicated in hypotension) – Levosimendan (improves RV/PA coupling without increasing myocardial O_2_ consumption) – Epinephrine (potent chrontrope, may increase PVR)	DO_2_/VO_2_: 3–4
	Volume overload	Pharmacolgic diuresis, or renal replacement therapy	– CVP: 8–12 cm H_2_O with normal SBP – Minimize RV dilation – Normalize septal wall motion
	Altered gas exchange	Careful ventilator managament – lung protective ventilation – attention to Δ*P* and mechanical power – PEEP titration	– Minimize acidosis (PaCO_2_ < 48s mHg), – PaO_2_/FiO_2_ > 150
	Increased RV afterload	Lung protective ventilator management Prone positioning	
**Patients on ECMO**	**Problem**	**Approach**	**Goal**
	RV dysfunction	Serial echocardiographic monitoring	Evaluate for worsening
	RV worsening depsite therapies listed above	Unload RV – RVAD – VA ECMO	Decrease RV load Follow institutional protocols

NE, norepinephrine; PVR, pulmonary vascular resistance; DO_2_, oxygen delivery; VO_2_, oxygen consumption; RV, right ventricle; PA, pulmonary artery; CVP, central venous pressure; Δ*P*, driving pressure; RVAD, right ventricular assist device.

A relatively high level of positive end-expiratory pressure (PEEP) early in the post-cannulation phase of ECMO has been associated with greater survival. In one study of 168 ECMO patients at three separate institutions, *V_t_* was decreased on day #1 of ECMO support to below 3–4 ml/Kg while median PEEP was 12 ± 3 cm H_2_O (60). The authors found that lower PEEP after 3 days on ECMO was associated with higher ICU mortality, as was lower *V_t_*. Additionally, higher pre-ECMO ventilator plateau pressures were associated with increased mortality. The authors note that higher PEEP may reduce atelectasis, improve *V*_A_/*Q* matching especially with the ultralow *V_t_* used in this population. A high PEEP strategy should be balanced with the knowledge that when excessively high it can reduce RV preload and result in regional alveolar overdistention and increased PVR, especially in the patient with heterogeneous lung disease.

Prone positioning has been shown to have beneficial effects on the right heart. The proposed mechanism is that improved oxygenation results in a decrease in PVR and thus improved unloading of the RV. In a study of 42 adults with ARDS, pre- and post-proning echocardiography was performed to evaluate for RV dysfunction (61). Half of the patients had ACP with RV enlargement and septal dyskinesia. Proning was associated with a decrease in airway pressure and PaCO_2_, and in those patients with ACP proning was associated with a decrease in RV dilation and septal dyskinesia. Other studies have shown prone position improved oxygenation in patients with ARDS on ECMO but pulmonary vascular and RV function were not reported (62, 63).

### Pharmacologic management of RV dysfunction

Pharmacologic support of the failing RV may also improve overall cardiac function. Norepinephrine improves coronary perfusion and cardiac output with smaller increases in myocardial oxygen consumption than epinephrine (64). Experimental models have shown this pharmacologic approach produces a decrease in RV wall stress and RVEDP with improved RV stroke volume that is not matched by fluid expansion alone (64). Dobutamine and milrinone (a phopsphodiesterase-3 inhibitor) improve RV contractility (40). Inhaled nitric oxide (iNO) has not been shown to have significant effects on mortality or duration of mechanical ventilation in adults with ARDS. Furthermore, its prolonged use has been associated with renal complications (65). However, because of its known pulmonary vasodilatory effects, iNO may have application in selective patients with RV dysfunction (40). Levosimendan increases sensitivity of troponin C for ionized calcium and thereby improves contractility. While not yet studied in the ARDS ECMO population, its use has been examined in 35 patients with ARDS related to septic shock criteria (66). In this study, the use of levosimendan was associated with improved cardiac output, RV function SvO_2_, as well as decreased mPAP and PVR. Further study is required to understand the role of this potential adjunct.

### Extracorporeal management of RV dysfunction

When RV dysfunction develops or progresses after initiation of V-V ECMO, the underlying lung disease may be complicated by other pathologic conditions. Investigation for progressive causes of RV dysfunction should be undertaken. Further management for RV failure may include inotropic support (Table 1). If pharmacologic support proves inadequate, alternative ECMO support strategies should be considered. The addition of an arterial cannula to change from V-V ECMO to V-VA can allow for direct circulatory support in addition to oxygenation and ventilation. Patient selection and optimal timing of transition of ECMO mode has not yet been clarified. With V-VA ECMO, attention to the relative flow in the arterial and venous return cannulas is essential to ensure appropriate oxygen delivery. Echocardiography may help determine the optimal ratio of arterial-to-venous flows. If this alteration proves inadequate and further support is required, the venous return cannula can sometimes be converted to a drainage site so as to provide VV-A ECMO support.

In a review of the Extracorporeal Life Support Organization (ELSO) registry of 717 ECMO exposures from 2009 to 2013 in adults with ARDS who received inotropic or vasopressor agents prior to cannulation, 82.4%% received V-V ECMO and 4% required conversion to VA ECMO (38). Patients who remained supported on V-V ECMO had higher pre-ECMO MAP and PEEP while patients who ultimately required V-A ECMO had lower pH and blood pressure, and higher inotrope/vasopressor receipt. Survival to discharge was significantly higher in the V-V group (58.0% vs. 42.9%).

In lieu of altering the ECMO circuit, an alternative option to provide cardiac support while on V-V ECMO includes the addition of an intra-aortic balloon pump (IABP). Although this device operates on the systemic circulation side, the use of IABP has been shown to be helpful in patients with RV or biventricular failure, likely related to the significant ventriculoventricular interactions that affect RV function (18, 67). One approach commonly used in children is to create an atrial septostomy via percutaneous balloon atrial septostomy (59). This maneuver can decompress an overloaded right heart without affecting RV afterload. Few studies exist to evaluate this intervention in adults with ARDS.

Another approach to managing patients with ARDS and RV dysfunction is the use of a temporary RV assist device (RVAD) placed percutaneously into the pulmonary artery [Protek Duo (LivaNova, London, UK)] (68). A single center report of patients with ARDS from SARS-CoV-2 described an approach using V-V ECMO with this RVAD (69). Of the first 40 patients, survival to discharge reached 82.5% (70). By the time of their report in 2021, 136 patients treated according to this protocol had completed their hospital course with 67% survival to discharge (68). Thus, the survival in the 96 patients after the initial report was 60%. In a smaller single center report, 18 patients were treated with this ECMO-RVAD approach and were compared with 21 patients treated with invasive mechanical ventilation alone (71). In-hospital and 30-day mortalities were significantly lower in the ECMO patients (11.1% vs. 52.4%, *p* = 0.008 and 5.6% vs. 42.9%, *p* = 0.011).

**Figure 1:**
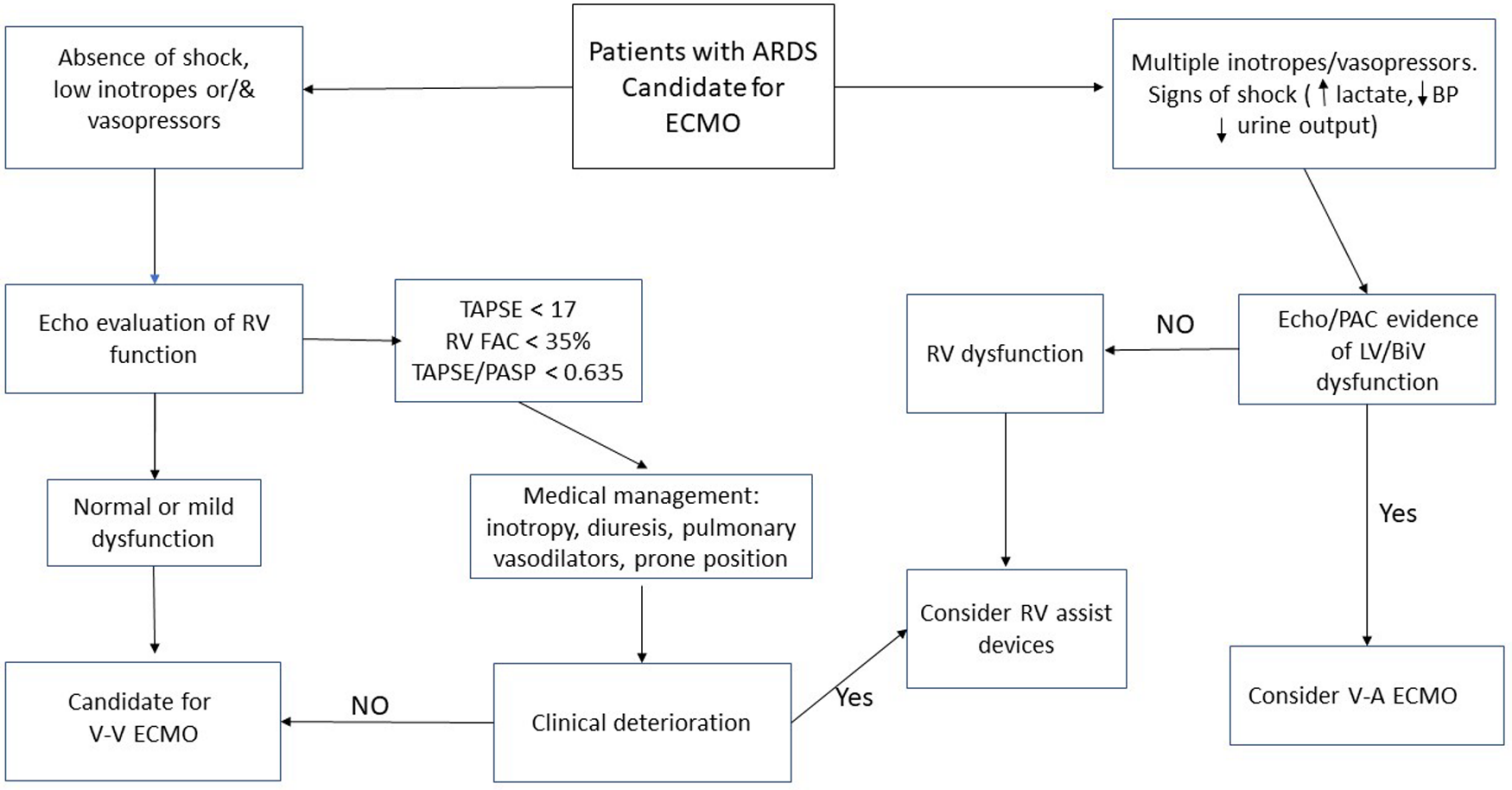
Approach to cannulation strategy in patients with ARDS and cardiac dysfunction.

Overall, the body of evidence supporting specific ECMO or RVAD approaches in the ARDS/RV dysfunction population is limited. There is a great need for randomized studies comparing different cannulation strategies in those with RV dysfunction and evaluating the optimal timing of RV support. One potential approach supplementary support is presented in Figure 1.

## Conclusion

Right ventricular and pulmonary vascular abnormalities occur commonly in patients with ARDS, especially those with disease severe enough to require V-V ECMO. Severe ARDS can result in elevated PVR thereby increasing RV afterload. This cascade of factors can result in RV dysfunction and subsequent LV dysfunction due to ventricular interdependence. RV dysfunction appears to be associated with worse outcomes in patients with ARDS. V-V ECMO may improve RV afterload by ameliorating hypercarbia and hypoxemia, while permitting protective or ultraprotective ventilation strategies. V-V ECMO usually provides adequate support for the RV but severe dysfunction has been documented despite ECMO support. Thus, care of the patient with ARDS and RV dysfunction on ECMO requires attention to optimizing right ventricular unloading even after cannulation. Careful echocardiographic evaluation should help guide management. Consideration of therapeutic approaches should be broad and include pharmacologic, ventilator, positional, and cannulation/VAD modalities. Further research is needed to elucidate conditions which may exacerbate RV dysfunction and to evaluate therapies which can optimize RV function in the patient requiring ECMO support.

## Author contributions

All authors listed have made a substantial, direct, and intellectual contribution to the work and approved it for publication.

## Conflict of interest

The authors declare that the research was conducted in the absence of any commercial or financial relationships that could be construed as a potential conflict of interest.
